# Epithelial Chloride Transport by CFTR Requires TMEM16A

**DOI:** 10.1038/s41598-017-10910-0

**Published:** 2017-09-29

**Authors:** Roberta Benedetto, Jiraporn Ousingsawat, Podchanart Wanitchakool, Yong Zhang, Michael J. Holtzman, Margarida Amaral, Jason R. Rock, Rainer Schreiber, Karl Kunzelmann

**Affiliations:** 1 Physiological institute, University of Regensburg, University street 31, D-93053 Regensburg, Germany; 2 Department of Medicine and Department of Cell Biology, Washington University School of Medicine, St. Louis, Missouri, USA; 3 University of Lisboa, Faculty of Sciences, BioISI - Biosystems & Integrative Sciences Institute, Campo Grande, C8, 1749-016 Lisboa, Portugal; 4 Department of Anatomy, University of California, San Francisco, USA

## Abstract

Cystic Fibrosis Transmembrane Conductance Regulator (CFTR) is the secretory chloride/bicarbonate channel in airways and intestine that is activated through ATP binding and phosphorylation by protein kinase A, but fails to operate in cystic fibrosis (CF). TMEM16A (also known as anoctamin 1, ANO1) is thought to function as the Ca^2+^ activated secretory chloride channel independent of CFTR. Here we report that tissue specific knockout of the *TMEM16A* gene in mouse intestine and airways not only eliminates Ca^2+^-activated Cl^−^ currents, but unexpectedly also abrogates CFTR-mediated Cl^−^ secretion and completely abolishes cAMP-activated whole cell currents. The data demonstrate fundamentally new roles of TMEM16A in differentiated epithelial cells: TMEM16A provides a mechanism for enhanced ER Ca^2+^ store release, possibly engaging Store Operated cAMP Signaling (SOcAMPS) and activating Ca^2+^ regulated adenylyl cyclases. TMEM16A is shown to be essential for proper activation and membrane expression of CFTR. This intimate regulatory relationship is the cause for the functional overlap of CFTR and Ca^2+^-dependent chloride transport.

## Introduction

The cystic fibrosis transmembrane conductance regulator (CFTR) and the calcium-activated chloride channel TMEM16A (anoctamin 1) are the two major secretory anion channels in intestinal and airway epithelia and therefore provide the critical regulation of mucus hydration at these sites^1–4^. TMEM16A and a third anion channel, SLC26A9, have been shown to be upregulated and particularly relevant during airway inflammation and asthma^5,6^. TMEM16A and SLC26A9 attenuate airway inflammation in cystic fibrosis (CF)^7^, prevent mucus obstruction during airway inflammation and attenuate the intestinal obstructive phenotype in CF mice^6,8^. In CF, TMEM16A and its regulator CLCA1 have been proposed as potential drug targets to compensate for the abrogated CFTR function in CF patients, while in asthma it may help to solubilize excess inflammatory mucus which may otherwise lead to airway obstruction^9,10^.

Previous *in vitro* studies suggested a functional relationship between calcium-activated TMEM16A and cAMP-regulated CFTR by some unknown mechanism^11–13^. Inhibition of TMEM16A by activated CFTR was suggested, while others reported similar pharmacological and functional properties for both Ca^2+^ and cAMP-activated Cl^−^ currents^14–16^. A recent study in human airway epithelial cells suggested CFTR as the principal chloride secretory pathway for both cAMP and purinergic; i.e. Ca^2+^ enhancing agonists^17^. Similarly, muscarinic stimulation was shown to activate CFTR via increase in intracellular cAMP, and both Src and Pyk2 tyrosine kinases^18^. Collectively, these data suggest that CFTR may function as a chloride channel that is activated by both cAMP and Ca^2+^.

Earlier work showed that mice lacking expression of TMEM16A in the airways present with a CF-like lung phenotype, suggesting that TMEM16A is essential for chloride secretion and maintenance of the airway surface liquid in mouse airways^4,19^. However, these results were obtained in conventional TMEM16A-deficient mice that exhibit multiple organ failures, requiring studies being performed on compromised newborn pups. We therefore generated mouse lines in which TMEM16A expression was selectively deleted in intestinal villus and crypt epithelial cells (using *Vil1-Cre–TMEM16A* ^*flox/flox*^ mice) or ciliated airway epithelial cells (using *FOXJ1-Cre–TMEM16A* ^*flox/flox*^ mice). This approach allowed for the first studies of adult mice with TMEM16A deficiency and demonstrated that TMEM16A expression is responsible for the calcium-activated chloride anion current in the intestine and lower respiratory airways and is essential for CFTR function at both of these mucosal sites.

## Results

### Intestinal epithelial cell knockout of TMEM16A eliminates CFTR currents

**Figure 1:**
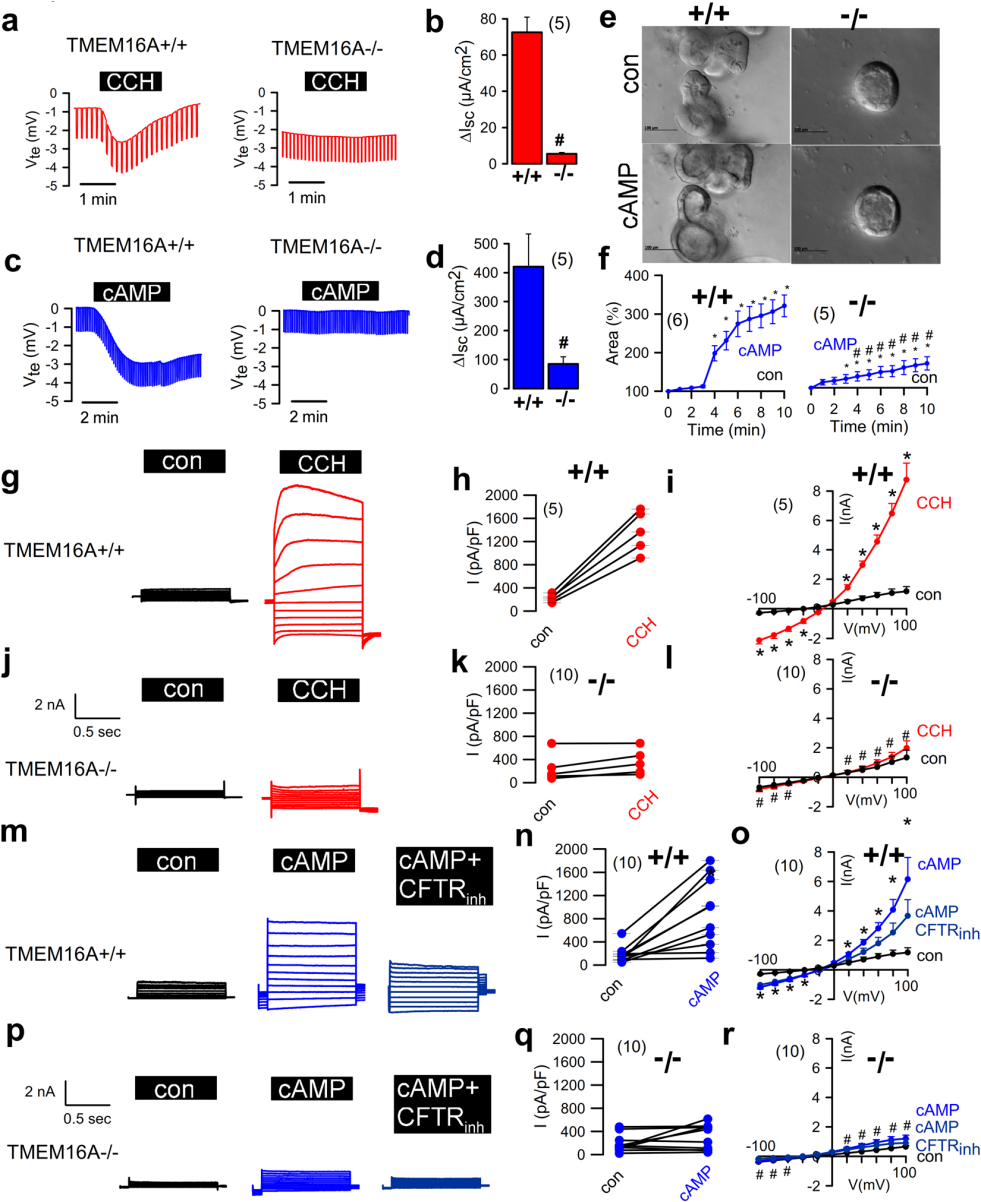
Intestinal epithelial knockout of TMEM16A eliminates CFTR currents. (**a**) Original recordings of the transepithelial voltage V_te_ and the effect of carbachol (CCH, 100 µM) in colonic epithelia from mice with intestinal epithelial knockout of TMEM16A (*Vil1-Cre–TMEM16A* ^*flox/flox*^; TMEM16A−/−) and wild-type mice (TMEM16A+/+). (**b**) Summary of the calculated CCH-induced short circuit currents (ΔI_sc_). (**c**,**d**) Original recordings of V_te_ and summary of cAMP (IBMX 100 µM/forskolin 2 µM)-induced ΔI_sc_ in +/+ and −/− colonic epithelia. (**e**,**f**) cAMP-induced secretion in intestinal organoids from +/+ and −/− intestines (upper panel) and summaries of luminal area increase (lower panel). (**g**–**i**) Activation of whole cell currents by CCH and inhibition by CaCCinhAO1 (AO1; 10 µM) in isolated intestinal epithelial cells from +/+ mice. Original recordings (**g**), individual experiments (**h**) and current/voltage relationships (**i**). (**j**–**l**) Corresponding experiments in intestinal epithelial cells from −/− mice. (**m**–**o**) Activation of whole cell currents by cAMP and inhibition by CFTRinh172 (CFTRinh; 10 µM) in isolated intestinal epithelial cells from +/+ mice. (**p**–**r**) Corresponding experiments in intestinal epithelial cells from −/− mice. Mean ± SEM; *Significant activation by cAMP or CCH (paired t-test). ^#^Significant difference between −/− and +/+ (unpaired t-test). (number of mice or cells, respectively).

An intestinal epithelial cell-specific *TMEM16A* gene knockout mouse (*Vil1-Cre–TMEM16A* ^*flox/flox*^) was generated from *Vil1-Cre* and *TMEM16A* ^*flox/flox*^ mice (Fig. S1) to determine TMEM16A function in the adult mouse intestinal epithelium. In contrast to reports of conventional TMEM16A-defiicent mice^4,19^, the *Vil1-Cre–TMEM16A* ^*flox/flox*^ mice did not show any difference in birth rate or lifespan, or manifest any baseline intestinal abnormalities (including intestinal obstruction or change in faecal water content) compared to control TMEM16A wild-type (wt) mice (*Vil1-Cre–TMEM16A* ^*wt/wt*^) (Fig. S2). Micro-perfused Ussing chamber experiments were used to assess ion transport of colonic epithelium by determining transepithelial voltage (V_te_) under open circuit conditions and calculating equivalent currents (I_sc_). Perhaps as expected, carbachol-stimulated calcium-activated anion transport was no longer present in colonic epithelia from *Vil1-Cre–TMEM16A* ^*flox/flox*^ compared to *Vil1-Cre–TMEM16A* ^*wt/wt*^ control mice (Fig. 1a,b). In addition, and rather unexpectedly, cAMP-activated anion transport was also markedly attenuated in intestinal epithelia from *Vil1-Cre–TMEM16A* ^*flox/flox*^ mice (Fig. 1c,d). This decrease in transport function was not accompanied by any change in the level of CFTR expression in colonic epithelial cells based on TMEM16A western blotting (Fig. S1d). In contrast, the pattern of CFTR expression in colonic epithelium of *Vil1-Cre–TMEM16A* ^*flox/flox*^ mice was different, with clearly compromised apical CFTR expression (Fig. S1a).

cAMP (IBMX and forskolin) stimulation failed to expand the luminal cavity in intestinal organoids from *Vil1-Cre–TMEM16A* ^*flox/flox*^ mice compared to wild-type control mice, indicating that the defect in intestinal cAMP-activated anion transport in *Vil1-Cre–TMEM16A* ^*flox/flox*^ mice was accompanied by decreased secretion (Fig. 1e,f). Consistent with these results, both carbachol and cAMP stimulation of ion currents determined by whole cell patch clamping were lost in freshly isolated mouse colonic epithelial cells from *Vil1-Cre–TMEM16A* ^*flox/flox*^ mice compared to wild-type control mice (Fig. 1g–r). Ca^2+^ and cAMP activated currents were identified by blockade with inhibitors for TMEM16A (CaCC-AO1) and CFTR (CFTRinh172)^20,21^ (Fig. 1m,o–r; Fig. 3g,h). However, as indicated below crosstalk of both, Ca^2+^ and cAMP signalling pathways may lead to a cross-inhibition by both inhibitors. Together, the results show that calcium-activated as well as cAMP-stimulated CFTR-dependent chloride secretion in mouse intestinal epithelial cells depends on TMEM16A expression.

### Respiratory epithelial knockout of TMEM16A eliminates CFTR currents

**Figure 2:**
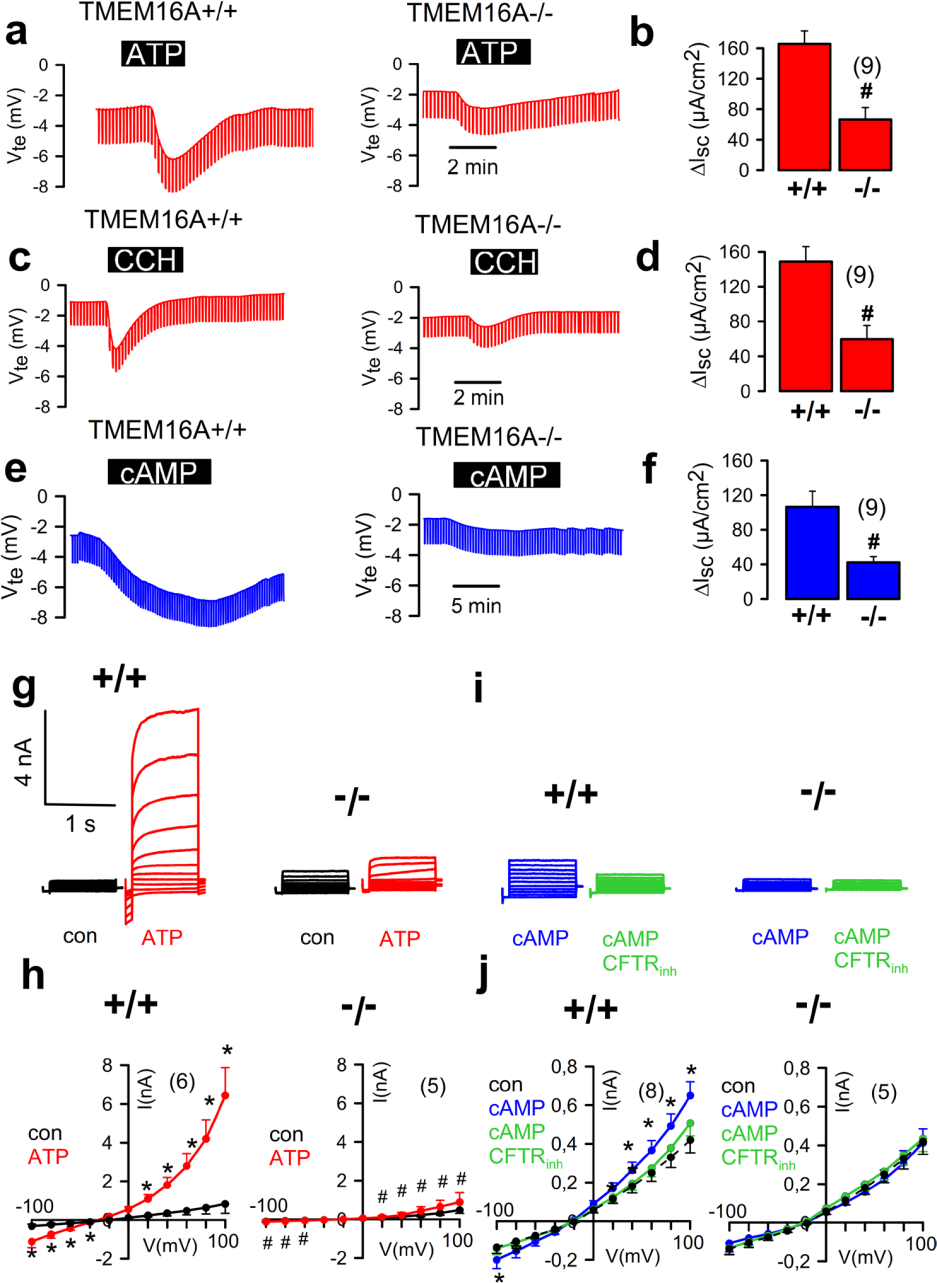
Respiratory epithelial knockout of TMEM16A eliminates CFTR currents. (**a**) Original recordings of the transepithelial voltage V_te_ and the effect of ATP (100 µM) in tracheas from *FOXJ1-Cre–TMEM16A* ^*wt/wt*^ (+/+) and *FOXJ1-Cre–TMEM16A* ^*flox/flox*^ (−/−) mice. (**b**) Summary of the calculated ATP-induced short circuit currents (ΔI_sc_). (**c**,**d**) Original recordings of V_te_ and summary of CCH-induced ΔI_sc_ in +/+ and −/− tracheas. (**e**,**f**) Original recordings of V_te_ and summary of cAMP (IBMX 100 µM/forskolin 2 µM)-induced ΔI_sc_ in +/+ and −/− tracheas. (**g**) Original recordings of whole cell currents activated by ATP in primary-cultured tracheal epithelial cells from TMEM16A+/+ and TMEM16A−/− mice. Experiments were performed in the presence of an inhibitor of Ca^2+^ activated K^+^ channels, TRAM-34 (100 nM). (**h**) Corresponding current/voltage relationships of whole cell currents activated by ATP in TMEM16A+/+ and TMEM16A−/− cells. (**i**) Original recordings of whole cell currents activated by cAMP in primary cultured respiratory epithelial cells from TMEM16A+/+ and TMEM16A−/− mice. (**j**) Corresponding current/voltage relationships of whole cell currents activated by cAMP in TMEM16A+/+ and TMEM16A−/− cells. Mean ± SEM; *Significant activation by cAMP or ATP (paired t-test). ^#^Significant difference between −/− and +/+ (unpaired t-test). (number of mice or cells, respectively).

We next examined the effect of TMEM16A-deficiency in respiratory airway function by generating an epithelial ciliated cell-specific knockout of the *TMEM16A* gene (*FOXJ1-Cre-TMEM16A* ^*flox/flox*^ mice) derived from *FOXJ1-Cre* and *TMEM16A* ^*loxp/loxp*^ mice (Figs 2 and S3a–c). TMEM16A was partially colocalized with CFTR in ciliated epithelial cells, but was absent in knockout airways (Fig. S3d,e). Notably, the TMEM16A antibody may have only detected larger expression levels. Like *Vil1-Cre-TMEM16A* ^*flox/flox*^ mice, the *FOXJ1-Cre-TMEM16A* ^*flox/flox*^ mice developed normally with birth rates and lifespans similar to wild-type control mice. Particle transport was assessed in isolated mouse tracheas as an index of mucociliary clearance (MC)^4^. It was not compromised, but unexpectedly even enhanced under basal conditions in tracheas from *FOXJ1-Cre-TMEM16A* ^*flox/flox*^ mice, compared to wild-type control tracheas. However, stimulation by ATP or carbachol did not further enhance MC in *FOXJ1-Cre-TMEM16A* ^*flox/flox*^ cells, in contrast to wild-type control cells (Fig. S2e). No mucus plugging was observed in the lungs of *FOXJ1-Cre-TMEM16A* ^*flox/flox*^ mice or wild-type control mice. Experiments with micro-perfused Ussing chambers showed that luminal ATP- or basolateral CCH-induced voltage deflections (ΔV_te_) and calculated ΔI_sc_ were both attenuated in tracheas from *FOXJ1-Cre-TMEM16A* ^*flox/flox*^ mice compared to wild-type control mice (Fig. 2a–d). As observed in cells from *Vil1-Cre-TMEM16A* ^*flox/flox*^ mice, also in tracheas from *FOXJ1-Cre-TMEM16A* ^*flox/flox*^ mice cAMP activated transport was inhibited (Fig. 2e,f). Moreover, whole cell patch clamp experiments on primary-cultured mouse tracheal epithelial cells demonstrated large ATP-activated Cl^−^ currents in cells from wild-type tracheas, which were absent in cells from *FOXJ1-Cre-TMEM16A* ^*flox/flox*^ tracheas, indicating that Ca^2+^ dependent Cl^−^ currents in mouse respiratory epithelial cells are entirely due to TMEM16A (Fig. 2g,h). cAMP activated whole cell Cl^−^ currents were small but detectable in wild-type airway cells, and were significantly inhibited by CFTRinh172. In contrast, in cells from *FOXJ1-Cre-TMEM16A* ^*flox/flox*^ tracheas no currents were activated by cAMP, and CFTRinh172 had no effects (Fig. 2i,j). Taken together, in both intestine and airways knockdown of TMEM16A strongly inhibited cAMP-activated, i.e. CFTR-related Cl^−^ transport in addition to the abolished Ca^2+^ dependent chloride secretion.

### Cl^−^ currents by CFTR and TMEM16A in human airway epithelial cells are linked

**Figure 3:**
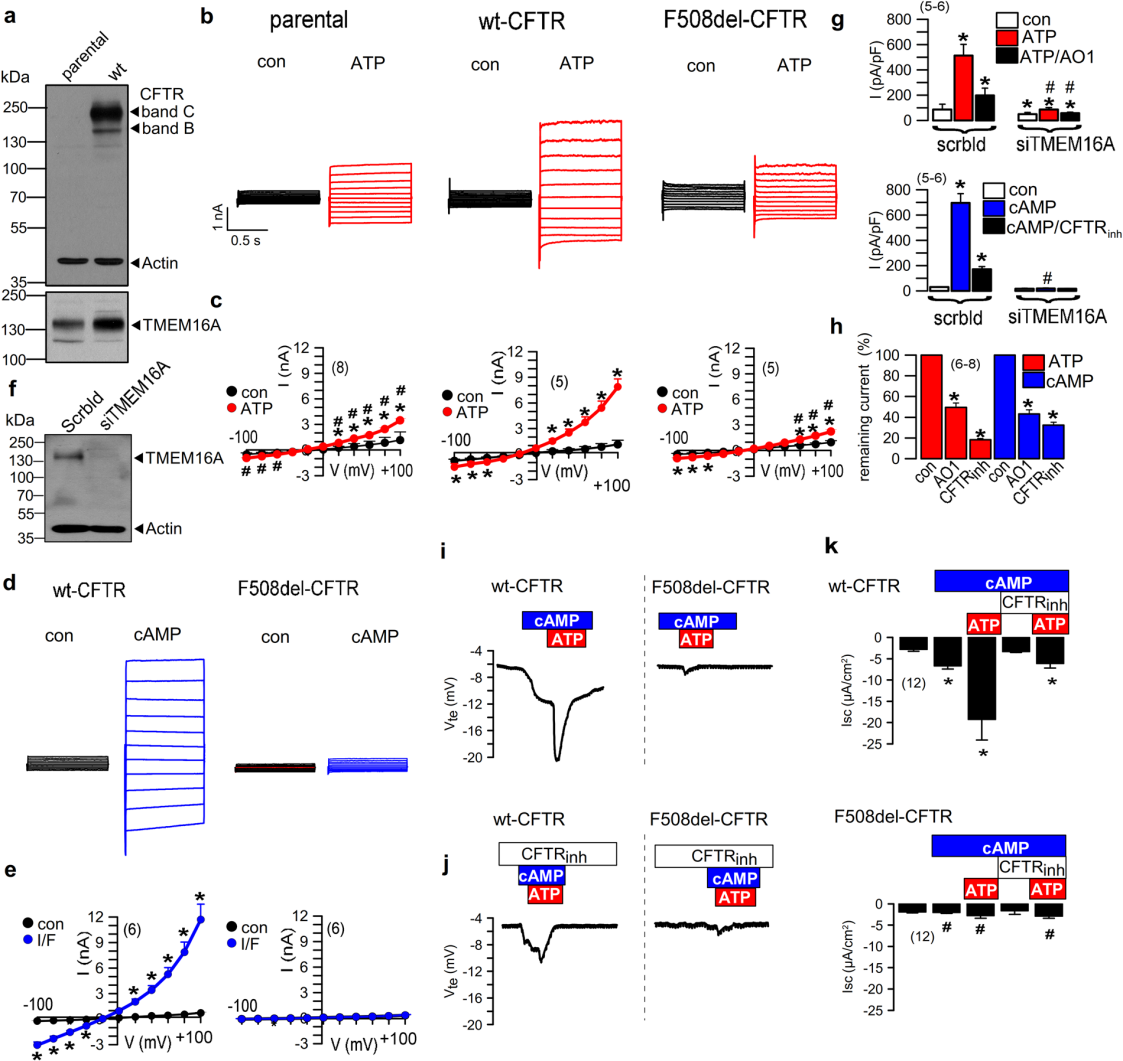
Cl^−^ currents by CFTR and TMEM16A in human airway epithelial cells cannot be strictly separated. (**a**) Western blot demonstrating wt-CFTR expression in CFBE/wt-CFTR cells, but not in CFBE parental cells. Both cells lines express similar levels of TMEM16A. (**b**) ATP (100 µM) -activated whole cell currents in CFBE parental cells and CFBE cells expressing wt-CFTR or F508del-CFTR. (**c**) Corresponding current/voltage relationships. (**d**) Whole cell currents activated by cAMP (100 µM IBMX/2 µM forskolin) in CFBE cells expressing wt-CFTR or F508del-CFTR. (**e**) Corresponding current/voltage relationships. (**f**) Western blot indicating suppression of TMEM16A in CFBE/wt-CFTR cells by siRNA (**g**) Summary of ATP (100 µM) -activated whole cell currents in CFBE/wt-CFTR cells treated with scrambled RNA or after siRNA-knockdown of TMEM16A (upper panel). Inhibition by CaCCinh-AO1 (10 µM). Summary of cAMP-activated whole cell currents in control (scrambled) and TMEM16A-knockdown cells, and effect of CFTRinh172 (10 µM). (**h**) Remaining currents after inhibition with CaCCinh_AO1 and CFTRinh172. Both blockers inhibit ATP- and cAMP-activated whole cell currents. (**i**) Transepithelial voltages recorded in polarized grown CFBE/wt-CFTR and CFBE/F508del-CFTR cells. Voltage deflections induced by cAMP or ATP. (**j**) Transepithelial voltages recorded in the presence of CFTRinh172 (10 µM). (**k**) Summaries of the corresponding calculated short circuit currents (I_sc_). Mean ± SEM; *Significant activation by ATP and cAMP, or currents inhibition by AO1 and CFTRinh, respectively (paired t-test). ^#^Significant difference between scrambled and siTMEM16A or between wt-CFTR and F508del-CFTR, respectively (paired t-test). (number of cells).

To translate the results obtained in mouse airways to humans, we studied cystic fibrosis bronchial epithelial (CFBE) cell lines engineered to stably express wt-CFTR or the most frequent mutant form F508del-CFTR, when compared to the parental cell line that does not express CFTR (Figs 3a and S4a)^22^. Although all three cell lines expressed comparable levels of TMEM16A (Fig. 3a, and S4a), the Ca^2+^-dependent whole cell currents activated by ATP were large in CFBE/wt-CFTR cells, but were small for CFBE/F508del-CFTR and parental cells (Fig. 3b,c). As expected cAMP-dependent currents were large in CFBE/wt-CFTR, but were essentially absent in CFBE/F508del-CFTR cells (Fig. 3d,e). Because large cAMP-dependent (CFTR) currents were paralleled by large TMEM16A currents, we knocked down *TMEM16A* gene expression in CFBE/wt-CFTR cells to see whether this affects CFTR currents (Fig. 3f). Strikingly, elimination of ATP-activated TMEM16A whole cell currents (Fig. 3g, upper panel), also abolished cAMP-activated CFTRinh172-inhibitable currents, despite expression of CFTR remained unaffected by TMEM16A-knockdown (Fig. 3g, lower panel, Fig. S4b). The results reproduce the functional interaction between TMEM16A and CFTR, and identify TMEM16A as the Ca^2+^ activated anion channel in human airway epithelial cells. There was also a considerable pharmacological overlap between CFTR and TMEM16A, as both currents were inhibited by the TMEM16A inhibitor AO1 and the CFTR blocker CFTRinh172 (Fig. 3h). Finally, when CFBE/wt-CFTR and CFBE/F508del-CFTR cells were grown to polarized epithelia on permeable supports and examined in Ussing chambers, the results were analogous: V_te_ and I_sc_ induced by either cAMP or ATP were large in CFBE/wt-CFTR epithelia, but were almost absent in CFBE/F508del-CFTR tissues. Moreover, CFTRinh172 blocked both CFTR (cAMP) and CaCC (ATP)-induced transport (Fig. 3i–k).

### TMEM16A activates CFTR by enhancing Ca^2+^ store release

**Figure 4:**
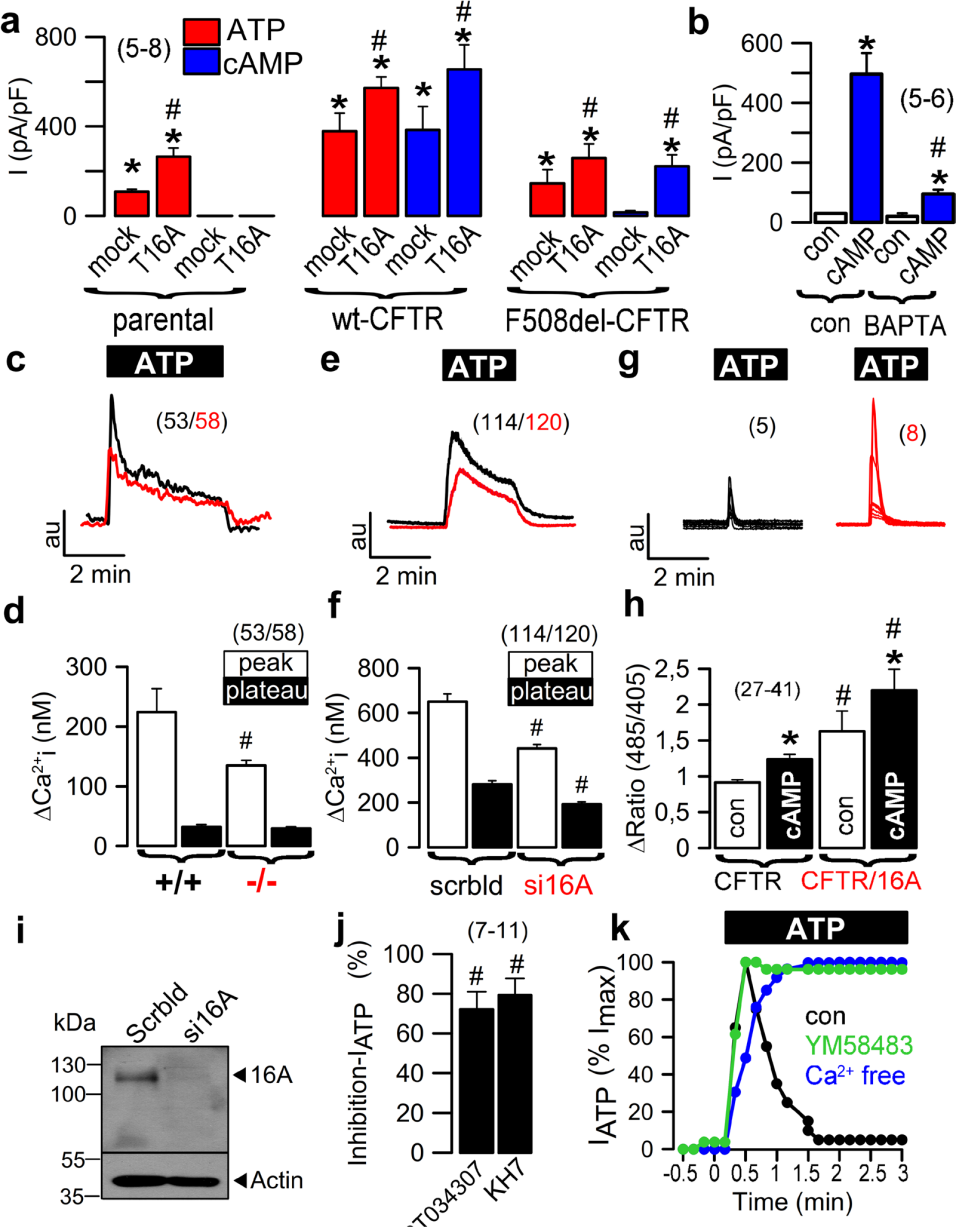
TMEM16A provides Ca^2+^ for activation of CFTR. (**a**) Summary of whole cell currents activated by increase in intracellular Ca^2+^ (ATP; 100 µM) and cAMP (IBMX 100 µM/forskolin 2 µM) in parental cells, CFBE/wt-CFTR and CFBE/F508del-CFTR cells, with or without (mock) additional expression of exogenous TMEM16A. Additional TMEM16A augments ATP-induced currents in all cell lines, enhances cAMP-activated currents in CFBE/wt-CFTR cells, and induces cAMP-activated currents in CFBE/F508del-CFTR cells (**b**) cAMP-activated whole cell currents in CFBE/wt-CFTR cells were inhibited by the Ca^2+^ chelator BAPTA-AM. (**c**,**d**) Mean recordings of ATP-induced rise in intracellular Ca^2+^ (Fura2) in primary airway epithelial cells from TMEM16A+/+ (black) and TMEM16A−/− (red) mice (upper panel). Summary of peak and plateau Ca^2+^ increase (lower panel). (**e**,**f**) Mean recordings of ATP-induced rise in intracellular Ca^2+^ (Fura2) in CFBE/wt-CFTR cells (upper panel) and summary of peak and plateau Ca^2+^, which were reduced after siRNA-knockout (red) of TMEM16A (lower panel). (**g**,**h**) Recordings of ATP-induced Ca^2+^ peaks in HEK293 cells expressing GCAMP2-tagged CFTR. The ATP-induced Ca^2+^ peaks are larger in TMEM16A (red) coexpressing cells (upper panel). Summaries of Ca^2+^ peaks in the absence or presence of cAMP (lower panel). (**i**) Western blot indicating knockdown of TMEM16A expression by siRNA. (**j**) Inhibition (in %) of ATP-activated Cl^−^ currents by two different inhibitors of Ca^2+^-dependent adenylate cyclases, ST034307 (30 µM) and KH7 (10 µM). (**k**) Time courses for activation of whole cell currents by ATP (100 µM) under control conditions, in the presence of the ORAI-inhibitor YM58483, and in the absence of extracellular Ca^2+^. Mean ± SEM; *Significant activation by ATP or cAMP (paired t-test). ^#^Significant difference when compared to mock, +/+, scrambled, absence of TMEM16A, or con, respectively (unpaired t-test). (number of cells or assays).

The present results establish that CFTR and TMEM16A currents are functionally linked and interdependent. To determine the mechanism for TMEM16A enhancement of CFTR activity, we analysed the effect of additional (exogenous) TMEM16A expression on CFTR function in CFBE/wt-CFTR cells. We found that additional TMEM16A enhanced ATP-activated TMEM16A currents in parental cells as expected, but in addition also enhanced cAMP-activated CFTR currents in CFBE/wt-CFTR and even in CFBE/F508del-CFTR cells (Fig. 4a). To determine the molecular mechanism for TMEM16A regulation of CFTR, we examined whether TMEM16A-driven release of ER store calcium might be responsible since TMEM16A is reported to enhance ER calcium store release^23–25^. This mechanism may cause CFTR activation in response to stimulation of purinergic and other phospholipase C coupled receptors^17,18^. We found that activation of wt-CFTR was markedly decreased when Ca^2+^ was chelated by BAPTA-AM (Fig. 4b). Moreover, release of Ca^2+^ from the ER store (peak) was significantly reduced in tracheal epithelial cells from *FOXJ1-Cre-TMEM16A* ^*flox/flox*^ mice (Fig. 4c,d). Correspondingly, ATP-induced Ca^2+^ store release was inhibited with siRNA-mediated knockdown of TMEM16A in airway epithelial cells (Fig. 4e,f). Notably, after knockdown of TMEM16A, a whole cell current could only be activated by cAMP in the presence of the Ca^2+^ ionophore ionomycin, confirming the role of Ca^2+^ and/or ER store release for activation of CFTR (Fig. S4c,d). To measure Ca^2+^ signals in close proximity of CFTR, the Ca^2+^ sensor GCAMP6 was fused to the C-terminus of CFTR and was expressed in HEK293 cells. TMEM16A enhanced ATP-stimulated Ca^2+^ release under control conditions and in the presence of cAMP (Fig. 4g,h).

### Role of Ca^2+^ regulated adenylate cyclases

Enhanced Ca^2+^ store release in the presence of TMEM16A may support activation of CFTR through Ca^2+^ dependent adenylate cyclases^17,26^. In support of this, we found that the IP_3_ receptor inhibitor xestospongin C markedly inhibited activation of CFTR by IBMX and forskolin (Fig. S4e), while the TMEM16A inhibitor CaCCinhAO1 (AO1) blocked basal and ATP-induced Ca^2+^ increase (Fig. S4f). AO1 also blocked cAMP-induced fluid secretion in organoids from T84 intestinal epithelial cells (Fig. S4g,h). Moreover, ST034307 and KH7, both inhibitors of Ca^2+^ dependent adenylate cyclases, potently inhibited ATP-activated Cl^−^ currents in CFBE/wt-CFTR cells (Fig. 4j). Because TMEM16A enhances ER Ca^2+^ store release, it is possible that compartmentalized increase in intracellular cAMP is induced by a mechanism recently identified as store operated cAMP signaling (SOcAMPs)^27^. SOcAMPs, i.e. increase of cAMP by ER store emptying, was shown to contribute to Ca^2+^-dependent activation of Cl^−^ secretion in T84 colonic epithelial cells^28^. Ca^2+^-refill of ER stores, was shown to terminate SOcAMPs, and occurs through store operated Ca^2+^ entry (SOCE) via ORAI channels. Remarkably, the ORAI channel blocker YM58483 or Ca^2+^ removal caused prolonged activation of Cl^−^ currents activated by ATP, which strongly suggests a contribution of SOcAMPs to activation of Cl^−^ secretion (Fig. 4k).

### Control of CFTR membrane expression by TMEM16A

**Figure 5:**
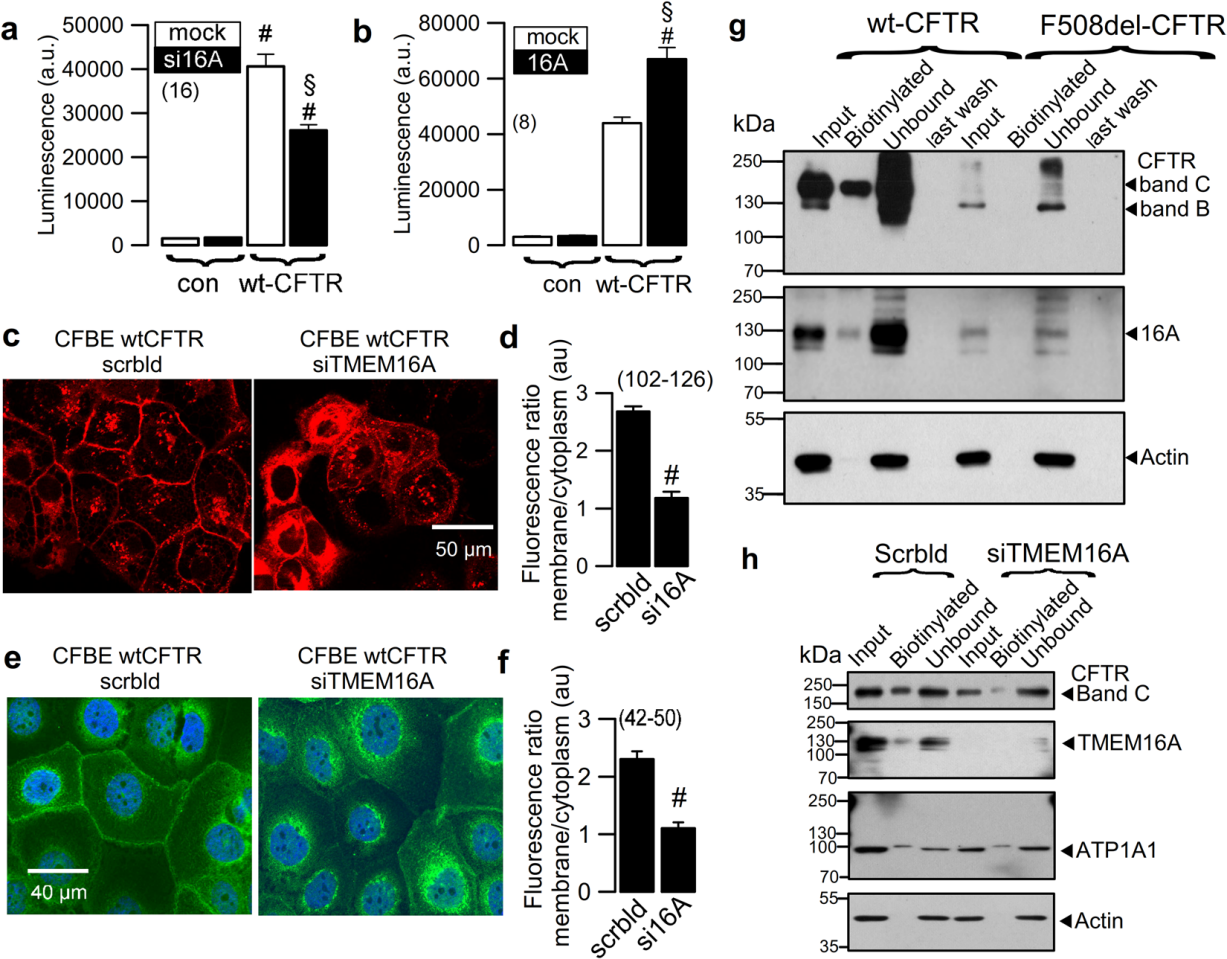
TMEM16A enhances membrane expression of CFTR. (**a**,**b**) Membrane expression of CFTR detected by chemiluminescence in CFBE/wt-CFTR cells expressing CFTR containing a FLAG tag in the first extracellular loop and a N-terminal cherry tag. Cells were exposed to a primary FLAG antibody (Sigma Taufkirchen, Germany, # F3165) and a secondary peroxidase-conjugated antibody. Luminescence was detected in CFBE/wt-CFTR cells but not in control parental cells (con). siRNA-knockdown of endogenous TMEM16A reduced chemiluminescence in CFBE/wt-CFTR cells (**a**), while additional expression of exogenous TMEM16A enhanced chemiluminescence (**b**). Very little background chemiluminescence was observed in the absence of CFTR (con). Mean ± SEM, (n) number of assays. ^#^Significant difference when compared to mock (unpaired t-test). (**c**,**d**) Life imaging of cherry-CFTR in CFBE/wt-CFTR cells with and without siRNA-knockdown of TMEM16A, as detected by cherry fluorescence. Summary of fluorescence intensity ratios (plasma membrane/cytosolic fluorescence) indicating redistribution of the fluorescence towards cytoplasm by siRNA-TMEM16A. (**e**,**f**) Antibody-staining of CFTR in CFBE/wt-CFTR cells with and without siRNA-knockdown of TMEM16A. Summary of fluorescence intensity ratios (plasma membrane/cytosolic fluorescence) indicating redistribution of the fluorescence towards cytoplasm by siRNA-TMEM16A. (**g**) Membrane biotinylation of CFBE/wt-CFTR and CFBE/F508del-CFTR cells, and detection of membrane and cytosolic fractions of CFTR and TMEM16A using Western blot. (**h**) Effect of TMEM16A-knockdown on biotinylation (membrane expression) of CFTR and TMEM16A. Mean ± SEM; ^#^Significant difference when compared to scrambled (paired t-test). (number of cells). Assays were performed in triplicates.

To further define the mechanisms for regulation of CFTR by TMEM16A, we examined membrane expression of CFTR. The results suggested a cellular mislocalization of CFTR in TMEM16A knockout tissues (Fig. S1a,b). We quantified the amount of plasma membrane CFTR in CFBE cells by chemiluminescence, using an extracellular FLAG epitope. Very little background luminescence was found in non-expressing parental cells (con), while a robust signal was detected in CFBE/wt-CFTR-FLAG cells (Fig. 5a). siRNA knockdown of TMEM16A (si16A) lowered membrane expression of CFTR (Fig. 5a), while additional expression of TMEM16A further enhanced luminescence (Fig. 5b). Cellular distribution of CFTR was analysed in CFBE cells in the presence or absence of TMEM16A. Membrane and cytosolic expression were quantified by analysing fluorescence intensities in the regions of interests (ROI) and are shown as proportions of membrane versus cytoplasmic fluorescence. CFTR was detected either by cherry fluorescence (Cherry-CFTR) in live imaging (Fig. 5c,d), or by using an anti-CFTR antibody in fixed cells (Fig. 5e,f). Both methods supplied comparable results and showed a shift of CFTR from cell membrane towards a cytosolic perinuclear localization. Using cell membrane surface biotinylation, we found membrane expression of CFTR and TMEM16A in CFBE/wt-CFTR cells, while neither CFTR nor TMEM16A could be biotinylated in CFBE/F508del-CFTR cells (Fig. 5g). Knockdown of TMEM16A in CFBE/wt-CFTR cells attenuated membrane expression of CFTR (Fig. 5h).

### Molecular interaction of CFTR and TMEM16A and a possible role of PDZ-interacting motifs

**Figure 6:**
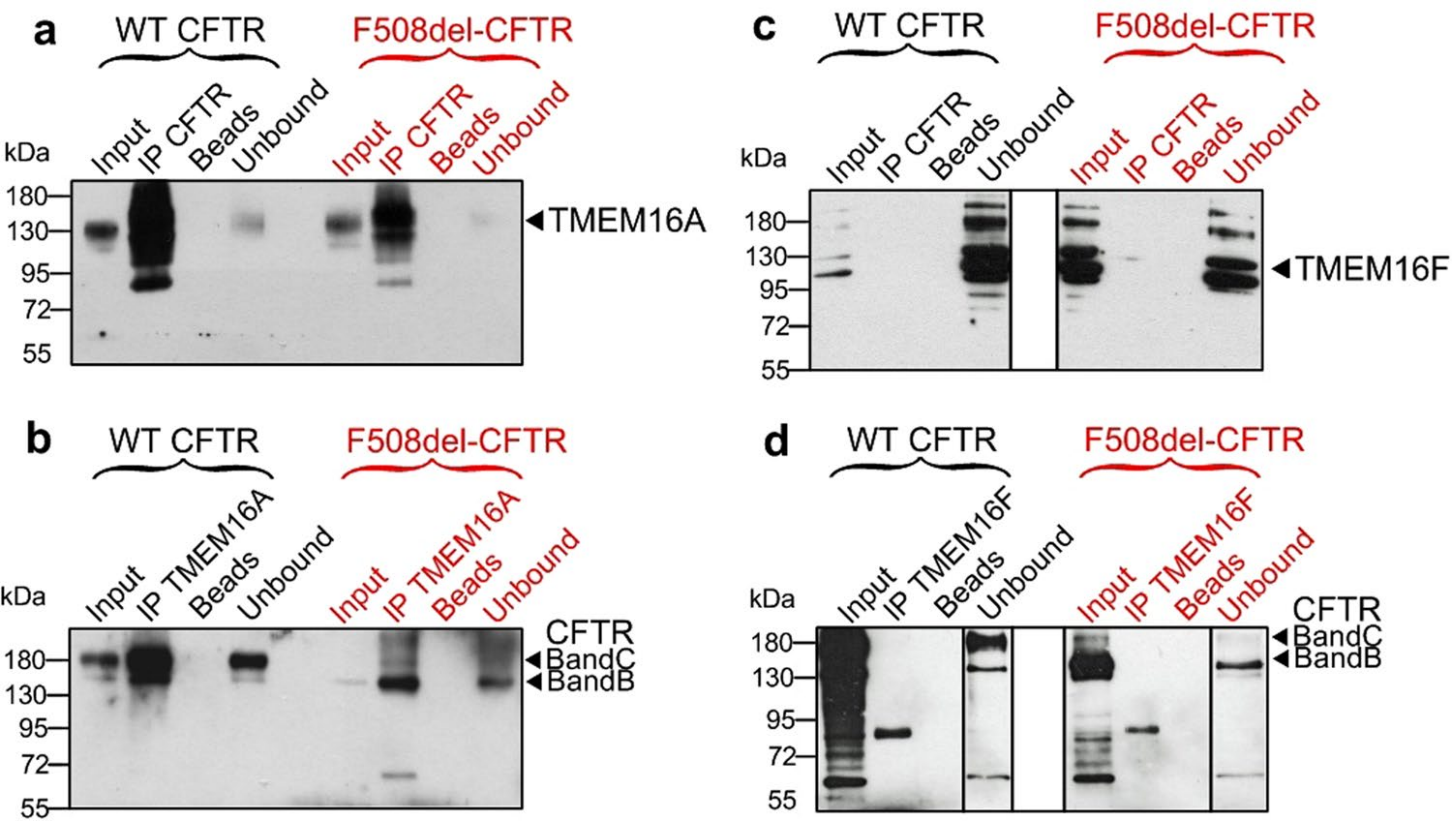
Molecular interaction of TMEM16A and CFTR. (**a**,**b**) Coimmunoprecipitation of CFTR and TMEM16A overexpressed in HEK293 cells. CFTR was pulled down by anti-CFTR antibody (Alomone labs, # ACL-006), while TMEM16A was pulled down by mouse monoclonal TMEM16A-antibody (Geneway GWB-MP178G). Both wt-CFTR and F508del-CFTR were found to interact with TMEM16A. Assays were performed in triplicates. (**c**,**d**) No coimmunoprecipitation was detected for CFTR and TMEM16F when experiments were performed under identical conditions using an TMEM16F antibody (Davids technology, Regensburg). White lines in the right blot indicate that lanes for unbound protein present on the same gel, have been relocated. Mean +/− SEM; ^#^Indicates significant difference (p < 0.05; unpaired t-test). (number of experiments).

The present results show a functional interaction of CFTR and TMEM16A. Both proteins may therefore be colocalized in a functional compartment or may even physically interact, possibly through adapter proteins like post-synaptic density protein/Drosophila disc large tumour suppressor/zonula occludens (PDZ) proteins^13^. In support of this, we found that wt-CFTR and F508del-CFTR could be coimmunoprecipitated with TMEM16A in CFBE cells (Fig. 6a,b). Notably, TMEM16A pulled down the fully glycosylated form of wt-CFTR (band C), and the core glycosylated form of F508del-CFTR (band B). Coimmunoprecipitation was not observed for wt-CFTR and the TMEM16A-paralogue TMEM16F (Fig. 6c,d). Molecular interaction may require the PDZ-interacting motifs present at C-terminus of CFTR and TMEM16A^29,30^. In support of this we found that deletion of PDZ-interacting motifs in either CFTR or TMEM16A reduced membrane expression of each protein (Fig. S5a,b). Membrane expression of both protein was further inhibited by simultaneous deletion of both PDZ-interacting motifs (Fig S5c). Taken together, control of CFTR through TMEM16A appears largely Ca^2+^ dependent, which also affects membrane expression of CFTR and may require anchoring of these proteins in a functional compartment by the help of PDZ proteins.

## Discussion

We demonstrate a complete absence of cAMP-activated Cl^−^ transport and a lack of Ca^2+^-dependent Cl^−^ secretion in large intestine and trachea from adult conditional TMEM16A knockout mice. A disturbed cAMP-dependent transport was not found in airways of conventional TMEM16A knockout pups^4,19^, confirming the somewhat different physiology of neonatal vs. adult airways^31^. Somewhat surprising the complete absence of Cl^−^ currents in TMEM16A knockout tissues did not cause any overt phenotype. Mouse airways lacking TMEM16A did not show any mucus accumulation, which may support the concept that airway Na^+^ transport is physiologically more relevant than Cl^−^ secretion in mouse airways^31^. The data show that most (Ca^2+^ and cAMP-dependent) murine airway Cl^−^ secretion depends on TMEM16A, with little contribution of CFTR^16,31^. This is somewhat surprising since TMEM16A is expressed at very low levels in (noninflamed) murine airways^5,32^.

Ruffin and coworkers found reduced TMEM16A-currents in mouse and human CF bronchial epithelium^33^. Our data obtained in human CFBE cells demonstrate a pronounced inter-dependence between CFTR and TMEM16A, in terms of membrane expression as well as activation of ion currents (Figs 4 and 5). Moreover, we were not able to discriminate clearly between CFTR and TMEM16A current based on ion channel inhibitors. The data demonstrate a remarkable overlap of cAMP and Ca^2+^-dependent signaling and are in line with studies reporting Ca^2+^-activated secretion through CFTR^17,18,26,34^ and cAMP-depending Ca^2+^ signaling controlling CFTR-mediated serous cell fluid secretion in porcine and human airways^35^.

Although ATP might be released during activation of CFTR and may activate P2Y_2_ receptors, this is unlikely to explain the present results, as activation of CFTR was not inhibited by the P2Y_2_ blocker suramin (100 µM), or by hydrolyzing extracellular ATP with apyrase (2 U/ml) (data not shown). In fact the present data demonstrate a mechanism through which TMEM16A facilitates local Ca^2+^ signals that are required for activation of apical CFTR (and probably basolateral K^+^ channels)^23–25^. Because TMEM16A facilitates ER Ca^2+^ store release, it may induce store-operated cAMP signaling^27^, which has been shown to control Ca^2+^ activated Cl^-^ secretion in T84 colonic epithelial cells^28^ (Fig. 4j). Moreover, further evidence is provided for a central role of Ca^2+^ activated adenylate cyclases^17^ (Fig. 4k).

In contrast to mouse airways expression of CFTR is pronounced in mouse large intestine, where it was found to be fully dependent on the presence of TMEM16A. Intestinal knockout of TMEM16A eliminated cAMP and Ca^2+^ activated Cl^−^ currents in colonic epithelial cells, again without causing intestinal obstructions. This is explained by the fact that cAMP-activated Cl^−^ currents were still present in the jejunum. Jejunal epithelial cells do not express TMEM16A and do not produce Ca^2+^ dependent, i.e. CCH induced Cl^−^ currents (Fig. S6)^31^. Jejunal epithelial cells obviously do not require TMEM16A for activation or membrane insertion of CFTR, which may suggest the role of another TMEM16 protein. Noteworthy, cAMP-activated currents were found to be reduced in jejunal epithelial cells from TMEM16K−/− mice^36^. Our results also explain Ca^2+^ dependent Cl^−^ and HCO_3_^−^ transport by CFTR in mouse intestine and other tissues^37–40^.

The present data suggest that TMEM16A is required for proper expression of CFTR in the plasma membrane (Fig. 5, Figs S1 and 3). Interaction of TMEM16A and CFTR in a functional signaling compartment at the plasma membrane may require the help of PDZ-proteins^41^ (Fig. S5c–f). TMEM16A has been shown to interact with IP3 receptors in a functional compartment also containing G-protein coupled receptors^25,42^. The present data add CFTR to such a compartment as it may be colocalized and interact directly or through PDZ proteins with TMEM16A. Due to the functional interaction of both proteins and cAMP/Ca^2+^-crosstalk, inhibitors for TMEM16A (e.g. CaCC-AO1) and CFTR (e.g. CFTRinh172) may be of limited use to dissect signalling pathways and the contribution of each channel to Cl^−^ transport in highly differentiated tissues^17,43^.

## Methods

### Animals, cells, isolation of crypts

All animal experiments were approved by the local ethics committee of the Government of Unterfranken/Würzburg (AZ: 55.2-2532-2-328) and were conducted according to the guidelines of the American Physiologic Society and the German law for the welfare of animals. Generation of *Vil1-Cre-TMEM16A* ^*flox/flox*^ mice and isolation of intestinal epithelial cells have been described earlier^44^. Knockout of TMEM16A in mouse airways way achieved by crossbreeding *Vil1-Cre-TMEM16A* ^*flox/flox*^ mice with FOXJ1-Cre transgenic mice generated as described previously^45^. Generation and culture of human cystic fibrosis bronchial epithelial cell lines (CFBE) is also described in previous reports^22,46^. CFBE cells have been originally generated by Dr. D.C.Gruenert (Cardiovascular Research Institute, UCSF, San Francisco, USA) in accordance with the local guidelines and regulations. Cells were grown in minimum essential medium supplemented with 2 mM glutamine and 2.5 µg/ml puromycin. For Ussing chamber measurements, the cells were grown on permeable supports (Corning® Costar® Snapwell™, Life Science, Tewksbury, MA). Respiratory epithelial cells were isolated from mice as described^47^ and were grown in AECGM plus supplement (Promocell, Heidelberg, Germany) that contained bovine pituitary extract 13 mg/ml, EGF 10 ng/ml, epinephrine 0.5 μg/ml, hydrocortisone 0.5 μg/ml, retinoic acid 0.1 ng/ml, transferrin 10 μg/ml, and triiodo-l-thyroxine 6.7 ng/ml. Media were further supplemented with 100 U/ml penicillin, 100 μg/ml streptomycin, 3 μg/ml fungizone, 50 μg/ml chloramphenicol, 0.1 mg/ml kanamycin. All media were supplemented with 10% fetal calf serum. Cells were incubated in 5% CO_2_ at pH 7.4. siRNA for hTMEM16A was transfected into CFBE cells using standard methods (Lipofectamine, Invitrogen, Darmstadt, Germany). Cells were studied 48 hrs after transfection. *cDNAs and RT-PCR*. For semi-quantitative RT-PCR, total RNA from mouse tracheal epithelial cells, crypts from mouse jejunum, proximal and distal colon and CFBE cells were isolated using NucleoSpin RNA II columns (Macherey-Nagel, Düren, Germany). Total RNA (1µg/50µl reaction) was reverse-transcribed using random primer (Promega, Mannheim, Germany) and M-MLV Reverse Transcriptase RNase H Minus (Promega, Mannheim, Germany). Each RT-PCR reaction contained sense and antisense primer (0.5 µM, see supplementary table 1), 0.5 µl cDNA and GoTaq Polymerase (Promega, Mannheim, Germany). After 2 min at 95 °C cDNA was amplified 30 cycles for 30 s at 95 °C, 30 s at 57 °C and 1 min at 72 °C. PCR products were visualized by loading on peqGREEN (Peqlab,VWR, Germany) containing agarose gels and analysed using Meta Morph Vers. 6.2 (Molecular Devices, USA). Human and mouse Primers for RT-PCR were (5′-3′, s-as): hTMEM16A CGACTACGTGTACATTTTCCG, GATTCCGATGTCTTTGGCTC; CFTR CATCTTTGGTGTTTCCTATG, GGAGTCTTTTGCACAATGG; mTMEM16A GTGACAAGACCTGCAGCTAC, GCTGCAGCTGTGGAGATTC; mCFTR GAATCCCCAGCTTATCCACG, CTTCACCATCATCTTCCCTAG; GAPDH GTATTGGGCGCCTGGTCAC, CTCCTGGAAGATGGTGATGG.

### Ussing chamber experiments

Isolated tracheas were put into ice cold bath solution containing (in mM/l) 145 NaCl, 0.4 KH2PO4, 1.6 K2HPO, 4.6 D-glucose, 1 MgCl2 1.3 Ca^2+^ gluconate; pH 7.4). After isolation of tracheas connective tissue was removed. Tissues were mounted into an Ussing chamber with a circular aperture of 0.785 mm^2^. Luminal and basolateral sides of the epithelium were perfused continuously at a rate of 5 ml/min. Luminal and basolateral solutions were heated to 37 °C, using a water jacket. Experiments were carried out under open-circuit conditions. Data were collected continuously using PowerLab (AD Instruments, Spechbach, Germany). Values for transepithelial voltages (Vte) were referred to the serosal side of the epithelium. Transepithelial resistances (Rte) were determined by applying short (1s) current pulses (ΔI = 0.5 µA). Rte and equivalent short circuit currents (Isc) were calculated according to Ohm’s law (Rte = ΔVte/ΔI, Isc = Vte/Rte).

### Intestinal organoids

The protocol for the small intestinal organoids isolation and culture was adopted from^48^. In brief crypts from the small intestine were isolated in Ca^2+^ free chelating buffer, and the crypts pellet was resuspended in Advanced DMDM/F12 (Thermo Fisher Scientific, Waltham, MA, USA) supplemented with 2mM glutathione, 100 U/ml penicillin, 100 μg/ml streptomycin, 10mM Hepes and mixed with ice- cold Matrigel^TM^ (Corning® Matrigel® Matrix, Life Sciences, Tewksbury, MA). The Matrigel^TM^ was overlayed with basal minigut media supplemented with Noggin and recombinant Rspo-1 (PeproTech, Hamburg, Germany).

### Patch Clamping

Primary-culture of mouse tracheal epithelial cells and CFBE cells were grown on coated glass cover slips for patch clamp experiments. Isolated intestinal crypts were immobilized on polylysine coated glass cover slips. If not indicated otherwise, patch pipettes were filled with a cytosolic-like solution containing in mM: KCl 30, K-gluconate 95, NaH_2_PO_4_ 1.2, Na_2_HPO_4_ 4.8, EGTA 1, Ca -gluconate 0.758, MgCl_2_ 1.03, D - glucose 5, ATP 3, pH 7.2. The Ca^2+^ activity was 0.1 µM. Coverslips were mounted in a perfused bath chamber on the stage of an inverted microscope (IM35, Zeiss) and kept at 37 °C. Transfected cells were identified by cotransfection of pIRES and antibody bound beads (Sigma Taufkirchen, Germany). The bath was perfused continuously with Ringer solution at a rate of 8 ml/min. Patch clamp experiments were performed in the fast whole cell configuration. Patch pipettes had an input resistance of 2–4 MΩ when filled with cytosolic like solution. Currents were corrected for serial resistance. The access conductance was monitored continuously and was 60–140 nS. Currents (voltage clamp) and voltages (current clamp) were recorded using a patch clamp amplifier (EPC 7, List Medical Electronics, Darmstadt, Germany), the LIH1600 interface and PULSE software (HEKA, Lambrecht, Germany) as well as Chart software (AD Instruments, Spechbach, Germany). Data were stored continuously on a computer hard disc and analyzed using PULSE software. In regular intervals, membrane voltage (*V*c) was clamped in steps of 20 mV from −100 to +100 mV from a holding voltage of −100 mV. Current density was calculated by dividing whole cell currents by cell capacitance.

### Measurement of [Ca^2+^]_i_

The plasma membrane bound calcium sensor GCaMP6 was fused to the N-terminus of CFTR. HEK293 cells grown on coated glass cover slips were transfected with GCaMP6-CFTR, and were mounted in a perfusion chamber 72 hrs after transfection. Cells were perfused with ringer solution at a rate of 8 ml/min at 37 °C. Cell fluorescence was measured continuously with an inverted microscope Axiovert S100 (Zeiss) using a x40 objective (Fluar 40x/1.3 Oil, Zeiss) and a high speed polychromator system (VisiChrome, Visitron, Puchheim, Germany). GCaMP6-CFTR was excited at 485 nm and 405 nm. Emission was recorded between 520 and 550 nm using a CCD-camera (CoolSnap HQ, Visitron). Control of experiments, imaging acquisition, and data analysis were done with the software package Meta-Fluor (Universal imaging, New York, USA). Alternatively, cells were loaded with Fura2 and intracellular Ca^2+^ concentrations were determined as described earlier^49^.

### Western Blotting, COIP, biotinylation

Protein was isolated from parental CFBE cells and from CFBE cells expressing wt-CFTR or F508del-CFTR using a sample buffer containing 50 mM Tris-HCl, 150 mM NaCl, 50 mM Tris, 100 mM dithiothreitol, 1% Nonidet P-40, 0.5% deoxycholate sodium, and 1% protease inhibitor mixture (Sigma, Taufkirchen, Germany). For co-immunoprecipitation CFBE cells (wt and dF508) were collected and lysed in 0.5% CHAPs lysis buffer containing 1X protease inhibitor cocktail. Protein (500 µg) was incubated with 6 μg of antibody (North American CFF, #596) and pre-cleaned protein G agarose (60 µl) on a rotator at 4 °C overnight. Afterward, beads were centrifuged and washed three times with 0.5% CHAPs lysis buffer containing 1X protease inhibitor cocktail. The Immunocomplexes were eluted by 2x sample buffer. For biotinylation CFBE cells (90–100% confluent) in T75 cm^2^ flasks were washed twice with ice-cold PBS (Ca^2+/^Mg^2+^), followed by incubation with Sulfo-NHS-SS-Biotin (Thermo Scientific, Waltham, MA USA) at 4 °C for 30 min according to the manufacturer’s instructions. Cells were lysed and homogenized in lysis buffer containing complete protease inhibitor cocktail (Roche, Penzberg, Germany) on ice for 30 minutes and centrifuged at 10,000g at 4 °C for 2 min. Biotin labelled surface proteins were captured on neutravidin agarose resin (Thermo Scientific, Waltham, MA USA) at room temperature for 1 h. The resins were washed 5 times with wash buffer containing protease inhibitor. Proteins bound to the resin were eluted with SDS PAGE sample buffer, and analysed by western blotting. Sample was separated by 8.5% SDS-PAGE and transferred to PVDF membrane (GE Healthcare, Munich, Germany). The membrane was blocked with 5% NFM/TBST or 5% NFM/PBST at RT for 1 h and incubated overnight 4 °C with rabbit polyclonal anti-TMEM16A AB (1:1000, 1% NFM/TBST), mouse monoclonal anti-CFTR AB (1:500, 3% NFM/PBST), rabbit polyclonal anti-Na+/K+-ATPase α AB (1:1000, 1% NFM/TBST) or mouse monoclonal anti β-actin AB (5% NFM/PBST). Subsequently, the membrane was incubated with HRP-conjugated donkey anti-rabbit or goat-anti mouse IgG at RT for 2 h. Immunoreactive signals were visualized using supersignal chemiluminescence substrate detection kit (Pierce Biotechonology, Rockford, USA).

### Chemiluminescence

CFBE wtCFTR and F508del-CFTR were tagged with an extracellular FLAG epitope and stably expression in CFBE cells using a doxycycline-inducible gene expression system. Surface CFTR expression was detected using monoclonal anti-FLAG M2-Peroxidase (Sigma, Taufkirchen, Germany) after 48 hr of doxycycline application. Cells were fixed in 4% paraformaldehyde, blocked in 5% BSA and incubated with anti-FLAG M2-Peroxidase (1:1,000). Chemiluminescene was detected using a SuperSignal West Pico chemiluminescence substrate (Thermo Scientific, Darmstadt Germany) and measured using plate reader NOVOstar (BMG Labtach, Offenburg, Germany).

### Immunocytochemistry

Mouse intestine, lungs and trachea were fixed by perfusion with 4% paraformaldehyde (PFA) and post-fixed in 0.5 mol/l sucrose, 4% PFA solution. Cryosections of 5 μm were incubated in 0.1% SDS for 5 min, washed with PBS, and blocked with 5% bovine serum albumin (BSA) and 0.04% Triton X-100 in PBS for 30 min. Sections were incubated with primary antibodies (Alomone Labs, #ACL-006) in 0.5% BSA and 0.04% Triton X-100 overnight at 4 °C and with Alexa Fluor 488 labeled donkey anti rabbit IgG (Invitrogen). Sections were counterstained with Hoe33342 (0.1 μg/ml PBS, Aplichem, Darmstadt, Germany). Immunofluorescence was detected using an Axiovert 200 microscope equipped with ApoTome and AxioVision (Zeiss, Germany). CFBE cells were grown on glass coverslips and fixed for 10 min with 4% (w/v) PFA at room temperature. After washing, the cells were permeabilized and blocked with 2% (w/v, PBS) bovine serum albumin and 0.04% (w/v, PBS) Triton-X-100 and incubated with primary anti-CFTR rabbit antibody (1:100, Alomone labs, # ACL-006) and anti-TMEM16A rabbit antibody (1:100, Geneway GWB-MP178G) over night at 4 °C. Binding of the primary antibody was visualized by incubation with a secondary antibody conjugated with Alexa 488 (Life Technologies, A-21206). Nuclei were stained with Hoe33342 (0.1 μg/ml PBS, Aplichem, Darmstadt, Germany). Cells were mounted on glass slides with fluorescent mounting medium (DAKO Cytomation, Hamburg, Germany) and examined with an ApoTome Axiovert 200 M fluorescence microscope (Zeiss, Göttingen, Germany).

### Materials and statistical analysis

All animal experiments were approved by local authorities and were conducted according to the guidelines of the American Physiological Society and the German law for welfare of animals. All compounds used were of highest available grade of purity. Data are reported as mean ± SEM. Student’s t-test (for paired or unpaired samples as appropriate) or ANOVA were used for statistical analysis. A p-value < 0.05 was accepted as significant difference.

All methods were carried out in accordance with guidelines and regulation. All experimental protocols were approved by the University of Regensburg and Government of Unterfranken. The senior author confirms that informed consent was obtained from all subjects.

## Electronic supplementary material

Supplementary information
