# Supplementary material for: Epithelial Chloride Transport by CFTR Requires TMEM16A
Source: Sci Rep. 2017 Sep 29;7:12397. doi: 10.1038/s41598-017-10910-0 (PMC5622110; doi:10.1038/s41598-017-10910-0)
Supplement: Supplementary file 1 — Supplementary information [file 41598_2017_10910_MOESM1_ESM.pdf]

Supplementary Information

Epithelial Chloride Transport by CFTR Requires TMEM16A

[1]Roberta Benedetto, [1]Jiraporn Ousingsawat, [1]Podchanart Wanitchakool,

[2]Yong Zhang, [2]Michael J. Holtzman, [3]Margarida Amaral, [4]Jason R. Rock

[1]Rainer Schreiber, [1]Karl Kunzelmann*

*corresponding author

[1]Physiological institute, University of Regensburg, University street 31, D-93053 Regensburg, Germany. Tel.: +49 (0)941 943 4302, Fax: +49 (0)941 943 4315

e-mail: karl.kunzelmann@ur.de

[2]Department of Medicine and Department of Cell Biology, Washington University School of Medicine, St. Louis, Missouri, USA

[3]University of Lisboa, Faculty of Sciences, BioISI - Biosystems & Integrative Sciences Institute, Campo Grande, C8, 1749-016 Lisboa, Portugal

[4]Department of Anatomy, University of California, San Francisco, USA

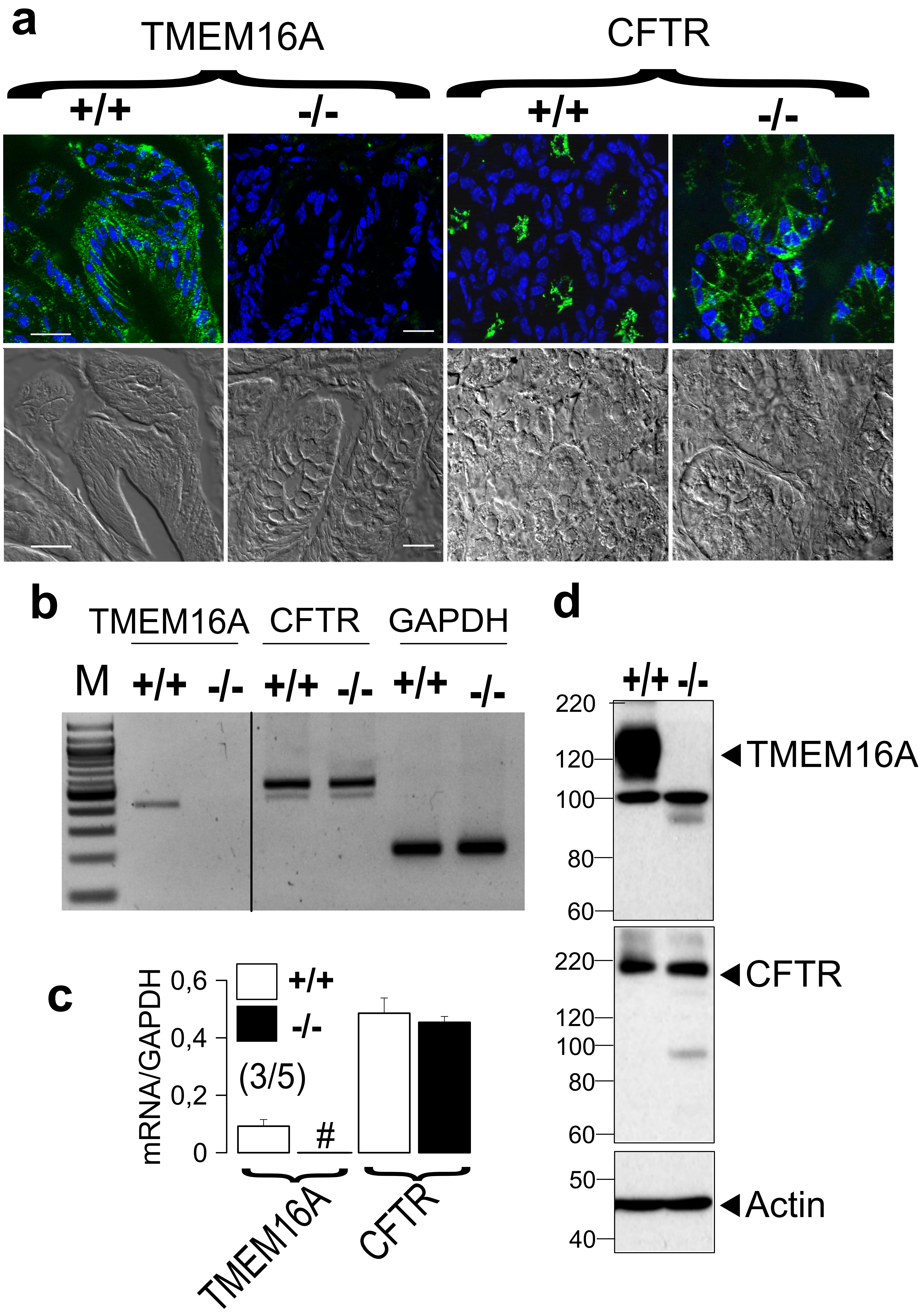

Supplementary Figure 1: *Expression of TMEM16A and CFTR in intestinal epithelial cells from Vil1-Cre–TMEM16A$^{wt/wt}$ (+/+) and Vil1-Cre–TMEM16A$^{flox/flox}$ (-/-) mice.* **a**) Immunocytochemistry of TMEM16A and CFTR in large intestinal epithelial cells from wild type mice (+/+) and mice with an epithelial cell specific knockout of TMEM16A (-/-) (upper panel). Differential interference contrast images of the tissues (lower panels). Note that deletion of TMEM16A caused a cellular redistribution of CFTR away from the apical and towards the lateral plasma membrane and cytosolic compartments. Bar indicates 20 µm. **b**) RT-PCR analysis of expression of TMEM16A and CFTR in isolated large intestinal epithelial cells. **c**) Semiquantitative analysis of mRNA expression (relative to GABDH). **d**) Western blots of TMEM16A and CFTR from isolated intestinal crypts of (+/+) and (-/-) mice. Mean ± SEM, (n) number of animals (wt/KO). #significant difference when compared to +/+ (unpaired t-test).

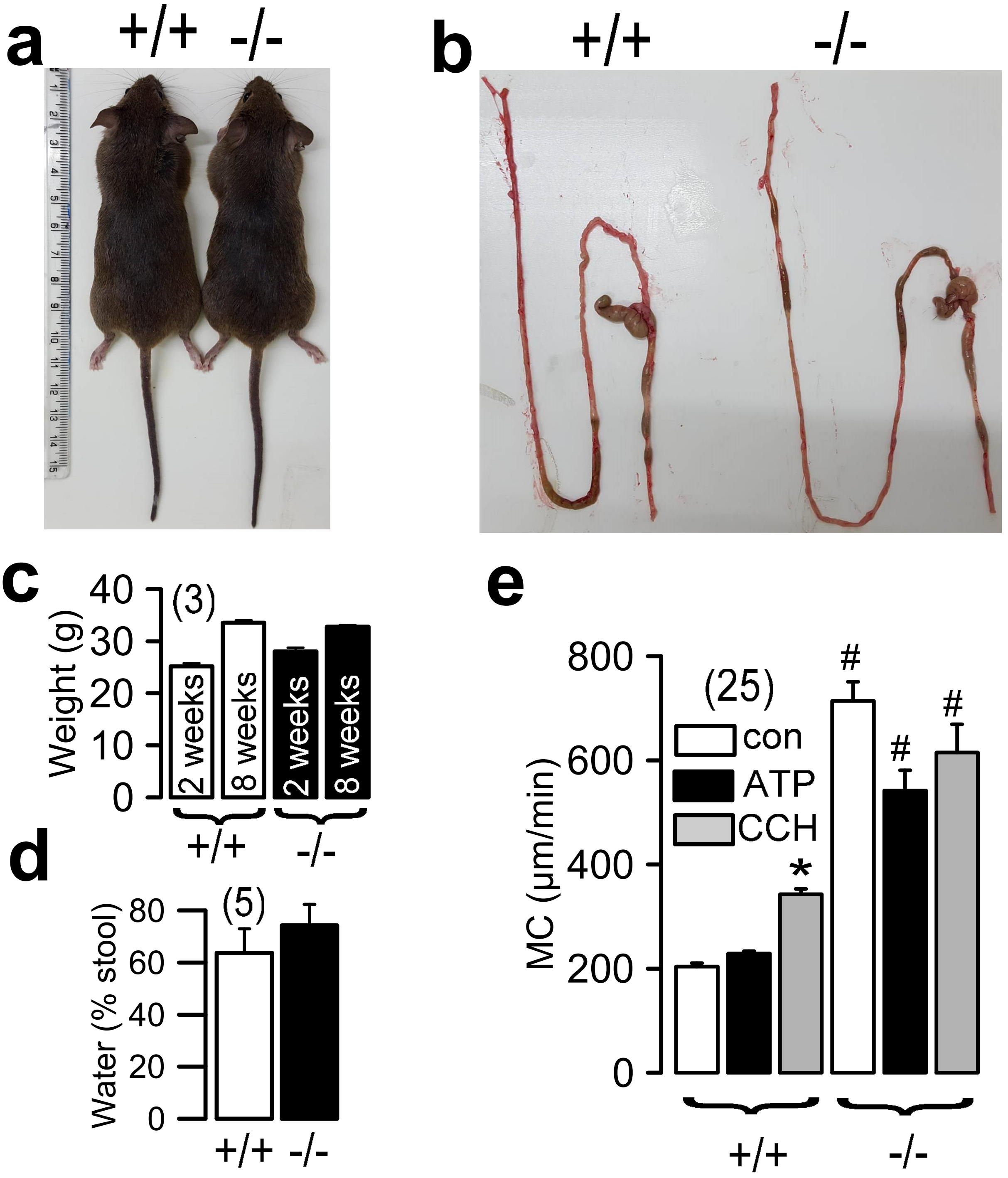

Supplementary Figure 2: *Lack of pathology in Vil1-Cre–TMEM16A$^{wt/wt}$ (+/+) and Vil1-Cre–TMEM16A$^{flox/flox}$ (-/-) mice.* **a**) Size of wild-type mice (+/+) and mice with an intestinal epithelial specific knockout of TMEM16A (-/-). **b**) Overall appearance of the intestine without obvious abnormalities in -/- mice. **c**) Weight increase in *(+/+)* and *(-/-)* mice. **d)** Percentage stool water content in samples from *(+/+)* and *(-/-) mice*. **e**) Mucociliary clearance (MC) measured by particle transport in isolated mouse tracheas placed in humidified chambers, as described earlier (Ousingsawat et al, 2009). MC was assessed under control conditions and after stimulation with 100 µM ATP (luminal) or 100 µM carbachol (basolateral). Mean ± SEM, (n) number of animals. *significant increase by CCH (paired t-test). $^{\#}$significant difference when compared to (+/+) mice.

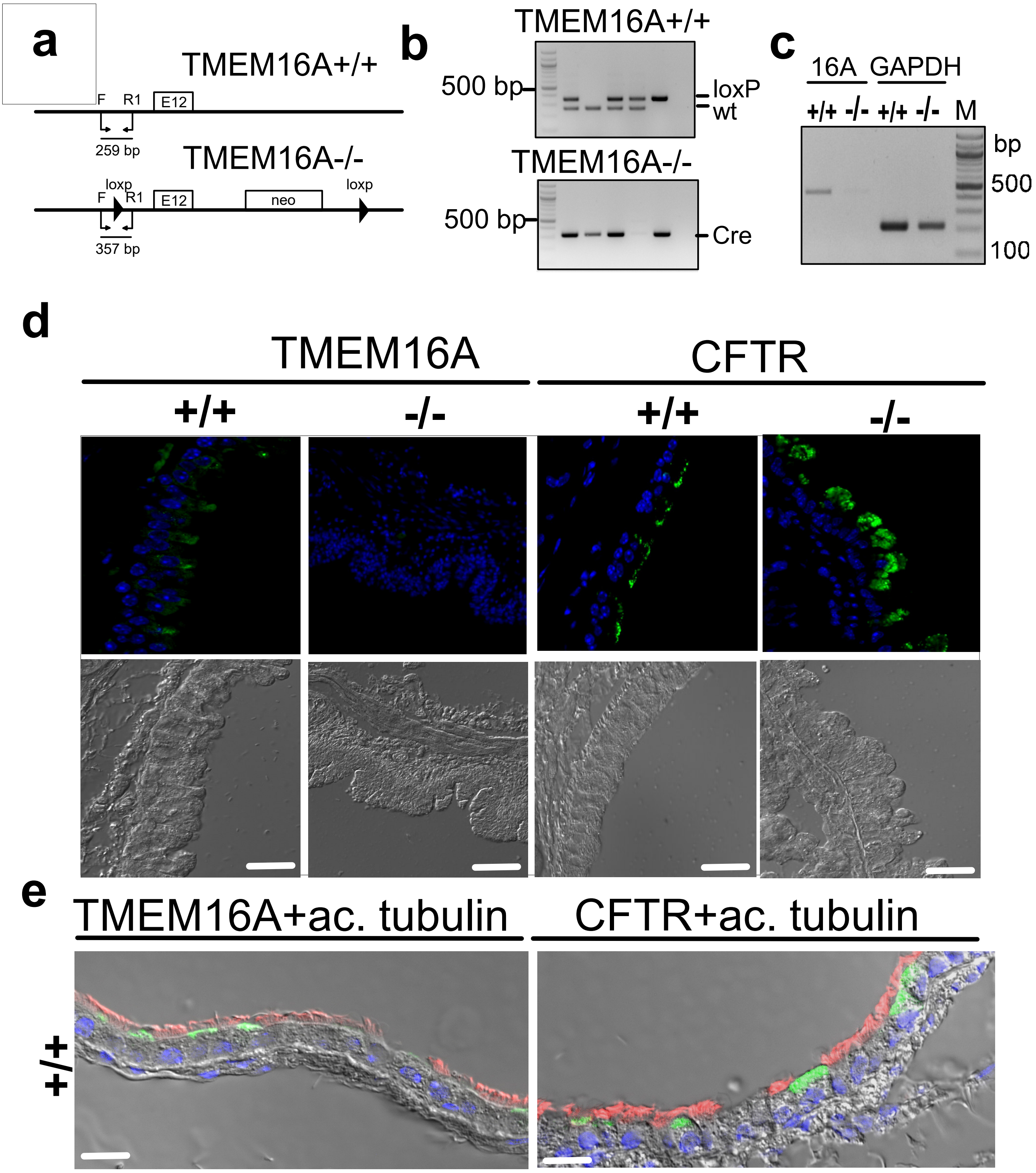

Supplementary Figure 3: *Expression of TMEM16A and CFTR in airway epithelial cells from TMEM16A+/+ and TMEM16A-/- mice.* **a**) Scheme indicating the loxP sites to generate -/- mice. **b,c**) Genotyping protocol and RT-PCR analysis of TMEM16A (16A) expression in epithelial cells. **d**) Immunocytochemistry of TMEM16A and CFTR in TMEM16A+/+ and TMEM16A-/- mice. Note the more diffuse staining of CFTR in TMEM16A-/-. **e**) Immunocytochemistry of TMEM16A/CFTR and acetylated tubulin suggesting heterogeneous expression of TMEM16A and CFTR and partial colocalization. Bar indicates 20 µm.

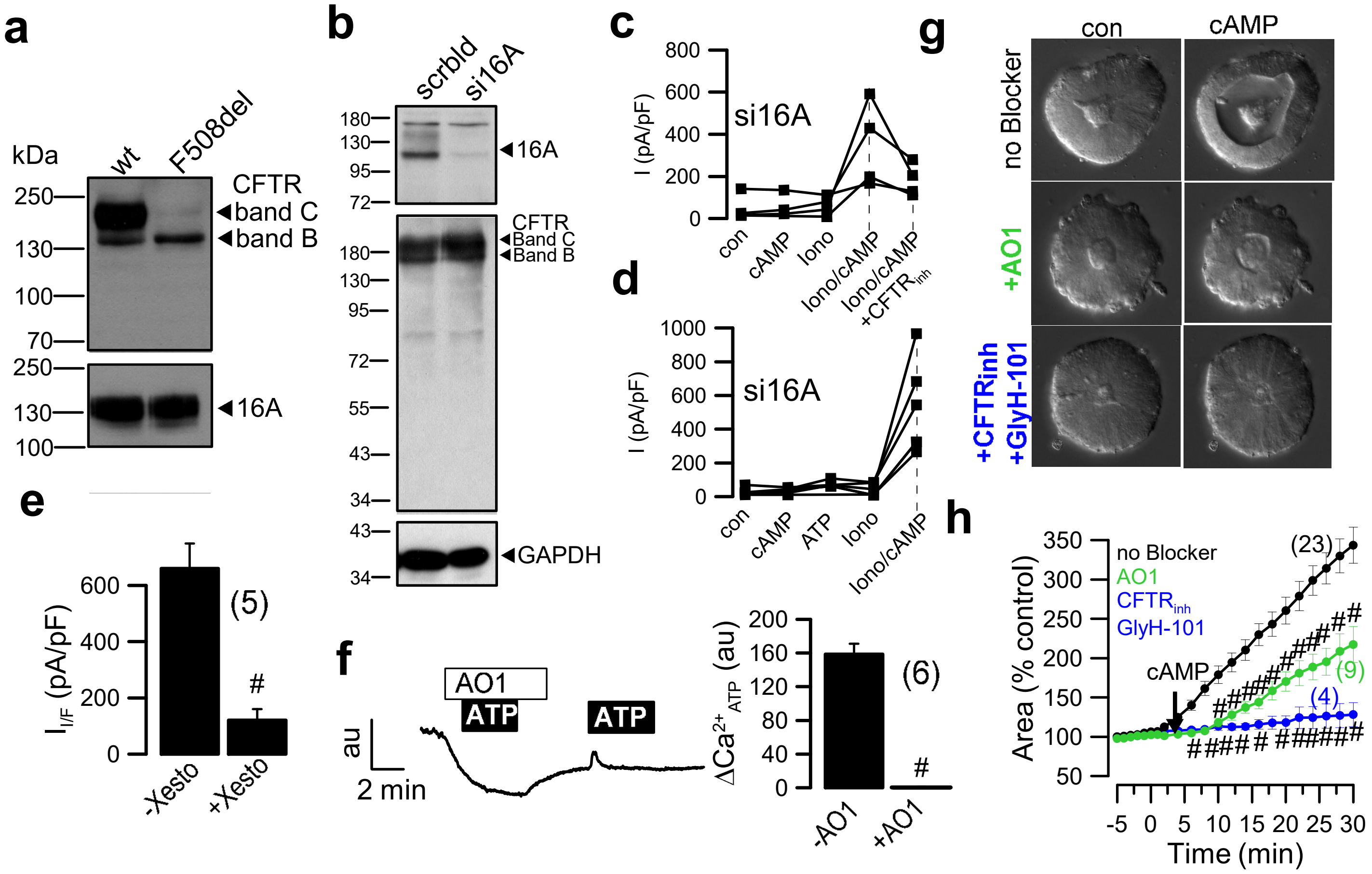

Supplementary Figure 4: *CFTR and TMEM16A (16A) dependent Cl^- transport cannot be separated.* **a**) Western blots of 16A and wt-CFTR or F508del-CFTR in stably expressing bronchial epithelial cells (CFBE). Expression levels for 16A are similar in both wt-CFTR or F508del-CFTR expressing cells. **b**) siRNA-knockdown of 16A (si16A) in CFBE cells does not affect expression of CFTR as shown by Western blotting. **c,d)** Continuous recording of whole cell current density in CFBE cells with siRNA-knockdown of 16A (si16A). Note that both IBMX/forskolin (100 µM/2 µM; cAMP) and ionomycin (**c**, 1 µM; Iono) or ATP (**d**, 100 µM) induced currents are inhibited by knockdown of TMEM16A (16A). In the presence of ionomycin, cAMP is able to activate whole cell currents, which are inhibited by $CFTR_{inh}$172. **e**) IBMX/forskolin (100 µM/2 µM) activated CFTR currents are inhibited by the IP_3R-inhibitor xestospongin (50 µM; Xesto). **f)** ATP (100 µM) induced Ca^{2+} transients as measured by GCAMP6 fused to CFTR, were completely suppressed by TMEM16A inhibitor AO1 (10 µM). Original recording (left) and summary of ATP induced Ca^{2+} changes (right). **g,h)** Organoids grown from T84 colonic epithelial cells demonstrate secretion upon stimulation with cAMP. Secretion is inhibited by AO1 (10 µM) as well as $CFTR_{inh}$172 (10 µM). Mean ± SEM, (n) number of cells. #significant difference when compared to absence of blocker (unpaired t-test).

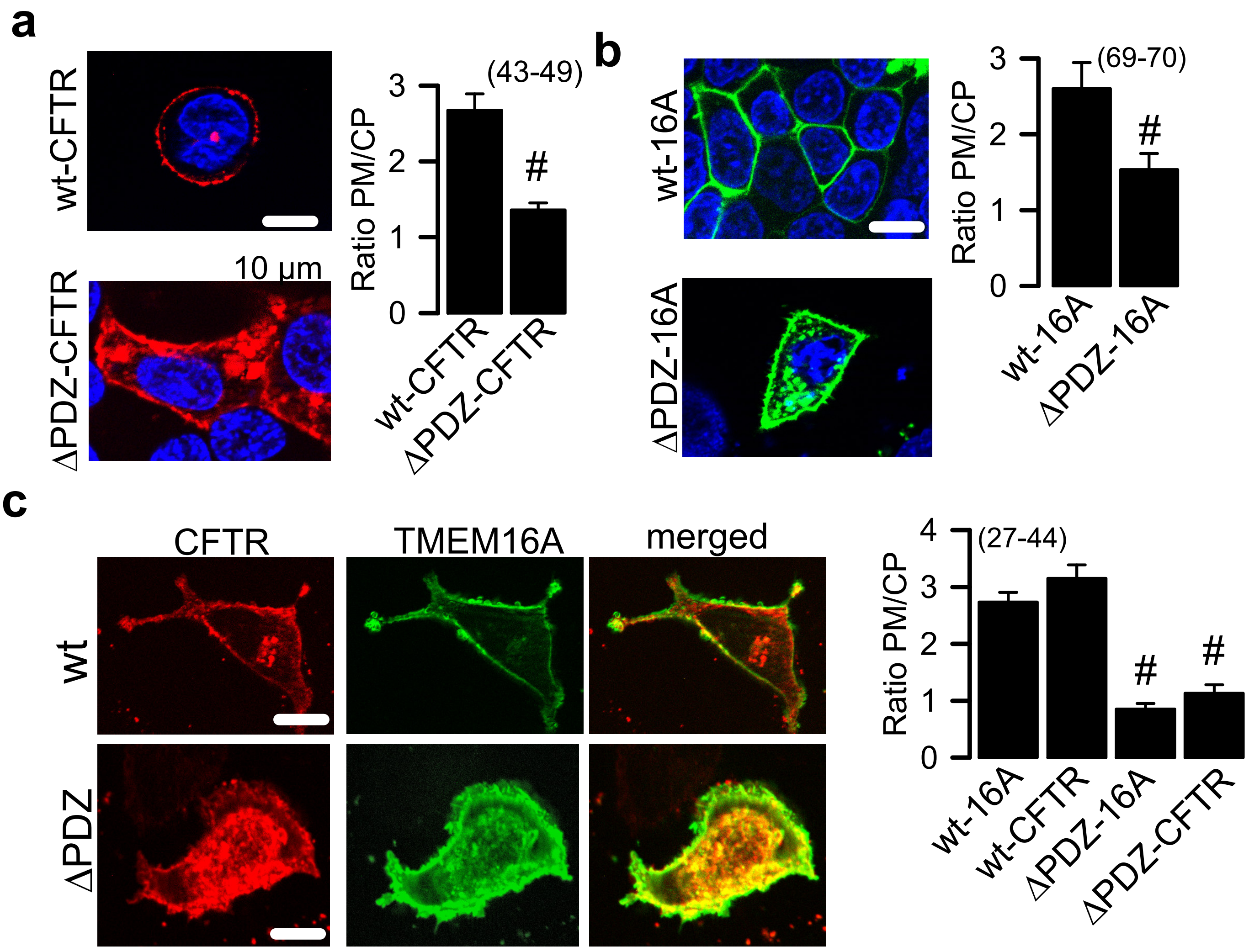

Supplementary Figure 5: *Plasma membrane expression of CFTR depends on TMEM16A (16A) containing a PDZ-binding domain.* (**a,b**) Immunocytochemistry of Cherry-CFTR and GFP-TMEM16A (16A) expressed in CFBE parental cells. Expression of wild type CFTR and TMEM16A (16A), respectively, or expression of truncated proteins, in which the C-terminal PDZ-binding motifs DTRL (ΔPDZ-CFTR) and GGVL (ΔPDZ-16A) were removed (Brannetti et al, Nucleic Acids Res. 31: 3709-11, 2003). Plasma membrane expression (PM) and cytoplasmic expression (CP) of wt and truncated proteins (ΔPDZ) were quantified by measuring fluorescence intensities in the region of interest, and ratios (PM/CP) were calculated. Plasma membrane expression of ΔPDZ-CFTR and ΔPDZ-16A were significantly reduced when compared to wt-proteins containing PDZ-binding motifs. (**c**) Coexpression of ΔPDZ-CFTR and ΔPDZ-16A leads to further decrease in membrane expression of CFTR and TMEM16A. Mean +/- SEM; #indicates significant difference compared to wt ($p<0.05$; unpaired t-test). (number of experiments).

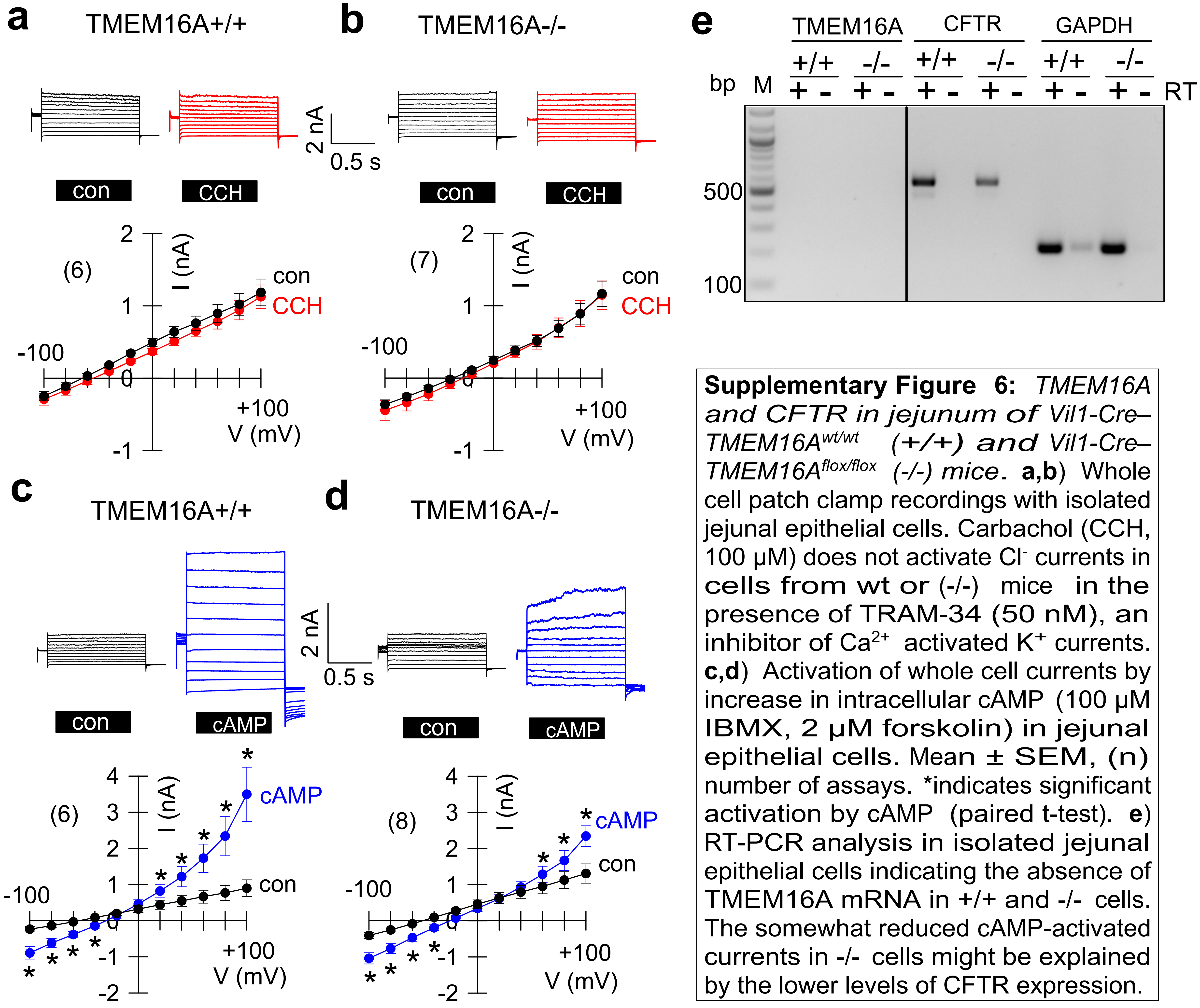

Supplementary Figure 6: *TMEM16A and CFTR in jejunum of Vil1-Cre–TMEM16A*$^{wt/wt}$ *(+/+) and Vil1-Cre–TMEM16A*$^{flox/flox}$ *(-/-) mice.* **a,b**) Whole cell patch clamp recordings with isolated jejunal epithelial cells. Carbachol (CCH, 100 µM) does not activate Cl^- currents in cells from wt or (-/-) mice in the presence of TRAM-34 (50 nM), an inhibitor of Ca^{2+} activated K^+ currents. **c,d**) Activation of whole cell currents by increase in intracellular cAMP (100 µM IBMX, 2 µM forskolin) in jejunal epithelial cells. Mean ± SEM, (n) number of assays. *indicates significant activation by cAMP (paired t-test). **e**) RT-PCR analysis in isolated jejunal epithelial cells indicating the absence of TMEM16A mRNA in +/+ and -/- cells. The somewhat reduced cAMP-activated currents in -/- cells might be explained by the lower levels of CFTR expression.
